# Why people hesitate to help: Neural correlates of the counter-dynamics of altruistic helping and individual differences in daily helping tendencies

**DOI:** 10.3389/fpsyg.2023.1080376

**Published:** 2023-03-14

**Authors:** Vidya Gani Wijaya, Kentaro Oba, Ryo Ishibashi, Motoaki Sugiura

**Affiliations:** ^1^Institute of Development, Aging and Cancer, Tohoku University, Sendai, Japan; ^2^School of Medicine and Health Sciences, Atma Jaya Catholic University of Indonesia, Jakarta, Indonesia; ^3^International Research Institute of Disaster Science, Tohoku University, Sendai, Japan

**Keywords:** altruism, empathy, decision-making, moral-dilemma, fMRI, personality, posterior cingulate cortex

## Abstract

Recent psychological and neuroimaging studies on altruism–egoism dilemmas have promoted our understanding of the processes underlying altruistic motivation; however, little attention has been paid to the egoistic counter-dynamics that prompt hesitancy to help. These counter-dynamics may involve the construction of reasons not to help based on contextual elaboration and explain individual differences in the tendency to help others in daily life. In this functional magnetic resonance imaging (fMRI) study, we explored the neural correlates of altruism–egoism dilemmas during empathy-driven helping decisions, with particular attention to the counter-dynamics related to individual helping tendency traits. We used two context-rich helping decision scenarios. In the empathy dilemma (Emp) scenario, empathy-driven motivation to help a poor person was associated with a cost, whereas in the economic-dilemma (Eco) scenario, self-beneficial motivation to help a non-poor person was associated with a cost. Our results showed activation of the right anterior prefrontal cortices, supramarginal gyrus, and posterior cingulate cortex (PCC) for the altruism–egoism dilemma (i.e., Emp > Eco). A significant negative effect of the helping tendency trait score was observed on PCC activation; interestingly, this effect was observed for both Emp and Eco dilemmas. The identified neural correlates of altruism–egoism dilemmas appear to be related to the construction of decision reasons based on contextual elaboration in naturalistic situations. In contrast to the classical view, our results suggest a two-stage model that includes an altruistic helping decision followed by counter-dynamics to determine the individual helping tendency.

## 1. Introduction

Although most people are happy to help others who are in adverse situations, we often hesitate to act after contemplating the consequences to ourselves. For example, we might easily decide to offer a bottle of water to a thirsty person, but then defer because we would have less to spend for lunch. We might offer organ donation to save a life, but then decline after considering our own medical risks and the concerns of close friends and family. Such non-helping decisions are typically accompanied by moral pain, which we must then overcome. Interestingly, some people often choose to help while others rarely experience such altruism–egoism dilemmas.

Recent studies on altruism–egoism dilemmas have focused primarily on altruistic motivation. Early studies cast doubt on the notion that helping behavior has a purely altruistic motivation (Harris, 1977; Schroeder et al., 1988; Cialdini, 1991). However, more recent research has been based mainly on the empathy–altruism hypothesis (Batson et al., 1991), in which altruistic helping of disadvantaged others is assumed to be driven purely by empathic feelings (Harbaugh et al., 2007; Rodrigue et al., 2011). Individual differences in the tendency to help others have also been attributed to differences in the tendency to show empathic concern (Batson et al., 1983; Decety et al., 2016).

Neuroimaging studies on altruism–egoism dilemmas have also focused on altruistic motivation; these studies have used an experimental paradigm in which participants can choose to help a person by “taking on” some of the pain that they are experiencing from electric shock (Singer et al., 2004). The anterior insula (AI) and temporoparietal junction (TPJ) are activated when a person observes another in pain; in this empathic response, the medial prefrontal cortex (mPFC) is activated at the point where the decision to help is made (Lamm et al., 2011; Feldman Hall et al., 2015). Activity in the AI and TPJ is greater in people who experience stronger empathic feelings when observing another person’s pain (Decety and Jackson, 2006; Decety and Lamm, 2007; Timmers et al., 2018). These findings have been replicated in individuals who tend to make altruistic decisions in economic games that do not involve pain (Sanfey, 2007; Cornelissen et al., 2011; Eimontaite et al., 2019).

In the past, psychologists were interested in the egoistic counter-dynamics that prompt hesitancy to help; early analyzes suggested that these dynamics may involve the construction of reasons not to help, based on contextual elaboration of the situation. A half century ago, psychologists discussed egoistic counter-dynamics in the context of the Kitty Genovese case, in which a woman was reportedly killed while being observed by 38 people. Although it is now known that witnesses did report the attack, many potential reasons were raised for witnesses not helping despite feeling empathy, including the awareness of other witnesses reducing the individual’s sense of responsibility, and the fear of being evaluated by others while asking for help (reviewed in, Latane and Darley, 1968). Such reasons would appear to be constructed based on contextual elaboration of the situation, allowing future simulation of potential cost and risk.

## 2. Literature review and hypotheses

### 2.1. The counter-dynamics of helping decision

Few experimental studies have addressed the counter-dynamics of helping decisions, possibly in part due to a preference for well-controlled experimental designs that prioritize real self-sacrifice in terms of monetary or physical cost while minimizing the context of the decision (Cutler and Campbell-Meiklejohn, 2019; Schaefer et al., 2021). Contextual elaboration in constructing reasons not to help is unlikely to occur in a minimal experimental design because a rich context is required to allow an individual to spontaneously explore reasons for not helping. In daily life, people may think that there are several “good” reasons for not helping. For example, they may think that help will be provided by other, more suitable people, or that helping might be misconstrued and viewed negatively by others (c.f. the case of Kitty Genovese). Moreover, it may be believed that there is a potential net negative effect of helping for society as a whole (where the time or money associated with helping could be used for other important purposes), or that there is a potential benefit for people in need of help, of solving their problems by themselves. Finally, it may be believed that the problems of people in need of help have arisen from their own behavior, such that it might be beneficial in the long run for them to take responsibility. Such egoistic counter-dynamics may share neural substrates with the resolution of moral dilemmas (Greene et al., 2004; Garrigan et al., 2016), which appear similar in terms of the contextual elaboration necessary to overcome the moral pain associated with deciding not to help. Several studies have investigated these neural substrates by devising various moral dilemmas, and have demonstrated involvement of the anterior prefrontal and lateral temporoparietal cortices, as well as the posterior cingulate cortex (PCC) (Moll et al., 2002, 2006; Greene et al., 2004; Reniers et al., 2012), which are the candidate neural substrates for egoistic counter-dynamics.

### 2.2. Individual differences in helping tendency

The scarcity of research on counter-dynamics is partly due to the lack of measures of individual differences in the tendency to help others in daily life (Moll et al., 2006; Volz et al., 2017; Piccinini and Schulz, 2019). Although some researchers have examined individual traits in altruistic motivation, most such studies have used the empathic concern subscale of the Interpersonal Reactive Index (IRI) (Davis, 1980) to evaluate empathy in association with particular types of decisions (Banissy et al., 2012; Paciello et al., 2013; Schaefer et al., 2021). Recently, an altruism subscale was developed for the Power to Live questionnaire, which measures eight personal characteristics associated with survival in disasters, identified through exploratory analyzes of interviews and questionnaire surveys of survivors of the 2011 Great East Japan Earthquake (Sugiura et al., 2015). The altruism subscale has been demonstrated to measure helping behavior during disaster evacuation at the expense of one’s own safety (Sugiura et al., 2020), and scores thereon may be inversely related to the tendency to recruit counter-dynamics when deciding whether to help.

### 2.3. Hypotheses development

In the current functional magnetic resonance imaging (fMRI) study, we aimed to identify the neural correlates of altruism–egoism dilemmas during empathy-driven helping decisions, with a particular focus on the counter-dynamics of helping decisions and individual tendencies to avoid helping. We used a context-rich scenario to allow contextual elaboration for constructing reasons to overcome moral pain associated with not helping. We used two helping decision scenarios, an empathy dilemma (Emp) scenario, in which empathy-driven motivation to help a disadvantaged other was associated with a cost, and an economic-dilemma (Eco) scenario, in which self-beneficial motivation to help a non-disadvantaged other was associated with a cost. Our hypothesis was that the decision as to whether to help would activate the moral dilemma network (i.e., anterior prefrontal and lateral temporoparietal cortices and the PCC) to a greater extent under the empathy dilemma than under the economic dilemma, predominantly in people who tend to help less in daily life. We used the altruism subscale of the Power to Live questionnaire as an index of the helping tendency.

## 3. Methods

### 3.1. Ethics statement

The protocol for this was reviewed and approved by the Tohoku University School of Medicine Ethics Committee (2018–1-785). All participants signed an informed consent form and were compensated for their participation. All participants were screened for fMRI contraindications and were given an orientation to the fMRI procedure prior to entering the scanner.

### 3.2. Participants

Forty healthy right-handed students in between July and August 2019 participated in the present study. All participants were undergraduate or graduate students of Tohoku University, Japan (26 males and 14 females; mean age = 21.2). All participants had no history of psychiatric condition, medical issue, or any of the standard contraindications to MRI scanning. Four participants were excluded from the analysis due to technical errors during data collection and three were excluded due to excessive head movement (> 6 mm).

### 3.3. Personal characteristics measurements

To measure individual tendencies of empathic concern and helping others in adverse situations, we used the empathic concern subscale of the Japanese version of the IRI (Himichi et al., 2017) and the altruism subscale of the Power to Live questionnaire (Sugiura et al., 2015), respectively, in association with stimulus and fMRI data analyzes. The empathic concern scale is composed of seven items, such as “I often have tender, concerned feelings for people less fortunate than me;” respondents rated the self-applicability of these statements on a 5-point scale (1: does not describe me at all, 5: describes me very well). The helping tendency (i.e., altruism) is indexed by five items: I like it when other people rely on me and are grateful to me; (2) When I see someone having trouble, I have to help them; (3) When someone asks me to do something for them, I cannot refuse; (4) Other people’s good fortune makes me happy so I like to help others; and (5) I am meddlesome and I like to do things for others; respondents rated the self-applicability of these statements on a 6-point scale (0: does not describe me at all, 5: describes me very well). We used the total score of all items for each scale (responses to the three reverse items for empathic concern were reverse-coded). The reliability (Cronbach’s α) and construct validity of the Japanese version of IRI (Himichi et al., 2017) and the Power to Live questionnaire have been established in disaster survivors (Sugiura et al., 2015) and in normal populations (Ishibashi et al., 2019; Matsuzaki et al., 2022). Using the current dataset, both of these instruments had a Cronbach’s α of 0.67.

### 3.4. Experimental tasks

**Figure 1:**
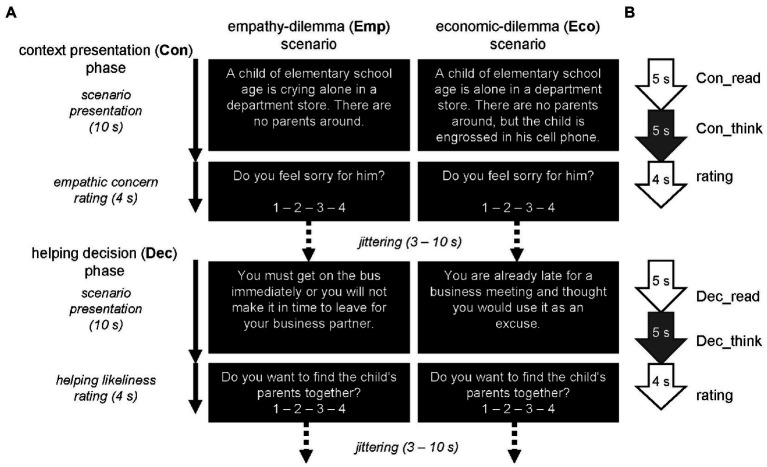
**(A)**: Experimental design. A pair of empathy-dilemma (Emp) and economic-dilemma (Eco) scenarios is shown as an example. **(B)**: Process model for fMRI analysis. Scenario presentation period was split in the middle and neural process in its latter half (thinking period) was compared between scenario types (i.e., Emp vs. Eco) separately for Con and Dec phases.

For both the Emp and Eco scenarios, each trial was composed of two phases: context presentation (Con) and helping decision (Dec) (Figure 1A). Each phase started with a presentation period (10 s) during which the scenario was described in detail in text format, followed by a rating period (4 s). In the Con phase, the scenario text described a situation introducing another person whom the participant would later decide whether to help or not; the person was in a disadvantaged situation in the Emp scenario but not in the Eco scenario. During the subsequent rating period, the participant was required to rate the degree of empathic concern they felt (“Do you feel empathy?”) toward the person using a 4-grade scale (1: not at all; 4: very much). In the subsequent Dec phase, the scenario text described a situation that would be relevant to a later helping decision. In the Emp scenario, possible reasons for deciding not to help include the presence of other people, being engaged in another important matter, and the belief that the person in need is responsible for their situation. In the Eco scenario, there were some potential benefits to the participant (e.g., monetary or social evaluation) that could mitigate the cost of helping a non-disadvantaged other. During the subsequent rating period, the participant was required to rate the likelihood of helping the person (“Are you likely to help?”) using a 4-grade scale (1: not at all; 4: very much). A total of 80 trials (40 per scenario type) were conducted; the order of the scenario types was pseudo-randomized. The interval between trials or phases varied between 3 and 10 s, while an eye-fixation cross was presented. The entire trial period was divided into four sessions, each of which lasted 769 s including 16-and 20 s rest periods at the beginning and end, respectively. Thus, the total length of the sessions was 51 min 16 s.

### 3.5. Stimuli

We prepared the Emp and Eco scenarios in pairs, such that each pair described an identical situation except for the key features manipulating empathic concern and the dilemma (Figure 1A). We collected the sample scenario pairs using an online cloud sourcing service (Lancers; Tokyo, Japan) and survey software (Qualtrics; Provo, UT, United States). We asked 100 applicants (without demographic specification or data) to create three pairs each after an explanation of the scenario specifications, and obtained 243 effective pairs. We created 82 candidate pairs using the situations and expressions in these 243 sample pairs, considering their appropriateness to the students, situational variability, and individual rating variability. Then, we selected 40 pairs for the fMRI experiment from these 82 candidates through an online experiment using the same cloud sourcing service and survey software. We asked 349 applicants (130 males and 219 females; mean age = 30.82 years) to perform the same tasks as the fMRI experiment (i.e., rating empathic concern and helping likelihood) for all 82 candidate pairs (i.e., 164 trials; presented in random order) and two personality trait measures. The 40 pairs were arbitrarily selected through discussion among the authors, with preference for situational variability across pairs, high correlation between empathic concern and trait empathic concern scores for EmpCon trials, and high correlation between helping likelihood and trait helping tendency scores for EmpDec trials.

### 3.6. Perspective instructions

Before entering the scanner, participants completed the empathy and helping likelihood personality trait questionnaires and practiced the fMRI task (20 trials, 10 scenario pairs not selected for the fMRI experiment) using a computer. They asked to consider themselves as a person who witness the situation that is written in texts and rated accordingly. In addition, all participants are asked to keep all of their belonging outside of the scanner room and participants who wear glasses are visually corrected using prepared glasses.

### 3.7. Experimental procedure

The participants asked to lay supine on the bed of the MRI scanner and stimuli were presented though a liquid-crystal display (LCD) monitor *via* a mirror attached to a head coil. Each participant performed the rating task by pushing the four buttons of an MRI-compatible response device (Current Designs, Philadelphia, PA, United States) with the first, second, third, and fourth fingers of their right hand. The assignment of the fingers to the buttons was counterbalanced across participants. The participant’s head was supported bilaterally by a cushion to reduce head motion, and they were instructed not to move their body throughout the experiment, except for the assigned finger. All trials were created, controlled, and recorded using the E-prime 2.0 software (Psychology Software Tools, Inc., Pittsburgh, PA, United States).

### 3.8. fMRI measurements

All MRI data were collected using a 3-T MRI scanner (Achieva Quasar Dual, Philips Medical Systems, Best, Netherlands). To obtain functional images of blood oxygenation level-dependent T2^*^-weighted MR signals, 40 transaxial images covering the entire brain were obtained using a gradient echo-planar imaging (EPI) sequence [repetition time (TR) = 2,500 ms; echo time (TE) = 30 ms; slide thickness = 3 mm; gap = 0 mm; flip angle (FA) = 85°; field of view (FOV) = 192 mm^2^; and scan matrix = 64 × 64]. High-resolution T1-weighted structural MR images were also obtained from each participant.

### 3.9. fMRI analysis

All functional images were analyzed using the Statistical Parametric Mapping software (SPM 12; Wellcome Department of Cognitive Neurology, London, UK) implemented in the MATLAB R2016a environment (MathWorks Inc., Natick, MA, United States). All analyzes were performed using the Montréal Neurological Institute (MNI) space. For pre-processing, head motion along the time-series EPI images was estimated and all images were realigned. Scanning time lags across the slices were corrected using a time series interpolation. The EPI images were spatially normalized to the MNI space using parameters estimated using the MNI-T1 template and structural T1 image of each participant, which were co-registered to the EPI image beforehand; a segmentation procedure was adopted to normalize the T1 image. Finally, all normalized EPI images were smoothed using a Gaussian kernel with a full width at half maximum of 8 mm.

A conventional two-level approach was applied to the multi-subject fMRI dataset for statistical analysis. At the first level, condition-specific hemodynamic responses were estimated at each voxel for each participant in a general linear model (GLM) framework. Each 10-s scenario presentation period of the two phases (Con and Dec) for the two scenario types (Emp and Eco) was split, with the first and second halves modeled separately as reading and thinking periods, with the latter being of interest in this study (Figure 1B). The rating periods of both phases in both scenario types were modeled together, coupled with a regressor in which the response magnitude was modulated parametrically with the rating score; these were intended to tease out sensorimotor effects across fingers. Thus, 10 condition-specific regressors (EmpCon_read, EmpCon_think, EmpDec_read, EmpDec_think, EcoCon_read, EcoCon_think, EcoDec_read, EcoDec_think, rating, and reting_parametric) were included in the model for each session. The six estimated head motion parameters were included to remove any artifacts caused by head motion. A high-pass filter (128 s cut-off) was adopted to remove low-frequency noise.

At the second level, between-subject statistical inferences were made for the contrasts of estimated condition-specific hemodynamic responses. To confirm the successful experimental manipulation of emotional concern in the Emp scenario, the contrast EmpCon_think > EcoCon_think was tested using a voxel-wise one-sample *t*-test; we expected higher activation of empathic concern-related regions during context presentation under the Emp than Eco scenario. Second, to identify the neural response characterizing the altruism–egoism dilemma during empathy-driven helping decisions, the contrast EmpDec_think > EcoDec_think was tested using a voxel-wise single-sample *t*-test.

Finally, to identify the neural correlates of individual differences in the helping tendency, we performed regression analysis using the helping tendency trait score (i.e., the altruism subscale of the Power to Live questionnaire). We conducted a voxel-wise search of the trait effect on two contrasts: EmpDec_think – EcoDec_think and EmpDec_think + EcoDec_think (i.e., against baseline). The former addressed neural responses specific to the empathic dilemma and the latter was common to both dilemma types. We also performed a *post-hoc* region-of-interest (ROI) regression analysis to address the trait effects for all identified activation peaks in these voxel-wise regression analyzes, as well as to those in the single-sample *t*-test of the contrast EmpDec_think > EcoDec_think. The ROI analysis addressed the trait effects separately for the EmpDec_think and EcoDec_think (i.e., against baseline) and the EmpDec_think – EcoDec_think contrasts.

The statistical threshold for the voxel-wise analysis was *p* < 0.001 (uncorrected) for the cluster formation, and corrected to family-wise error (*p* < 0.05) using cluster size, and assuming the entire brain as the search volume. For the one-sample *t*-test of the EmpCon_think > EcoCon_think contrast, small-volume correction was applied to empathic concern-related regions, i.e., the bilateral AI and right TPJ (Feldman Hall et al., 2015). Volume images for bilateral AIs were obtained from the Automated Anatomical Labeling (AAL) brain atlas (Tzourio-Mazoyer et al., 2002), while those for the right TPJ used a sphere with a 20-mm radius centered at [54, −54, 24] (Morishima et al., 2012). For *post-hoc* ROI analyzes, a 20-mm-radius spherical ROI from the Marsbar toolbox v0.44 was used (Brett et al., 2002), with a statistical threshold of *p* < 0.05 (uncorrected).

## 4. Results

### 4.1. Behavioral data

We compared the average rating scores and their correlations with two personality scores, between two scenario types. For the Con phase, we aimed to confirm a high empathic concern rating under the Emp scenario (i.e., close to the maximum of 4), and a low rating under the Eco scenario (i.e., close to the minimum of 1), to ensure that empathy manipulation was successful; positive correlation between the former and the empathic concern trait was also expected. For the Dec phase, we expected the average helping likelihood rating to be close to the midpoint of the range (2.5), reflecting a balance between helping and non-helping decisions among participants for both scenario types. However, we expected positive correlation between the rating and trait helping tendency only for the Emp scenario.

The results are summarized in Table 1. As expected, the average empathy concern rating was >3 for the Emp scenario and < 2 for the Eco scenario, with a mean difference close to 1.5 (*p* < 0.001, two-tailed, single-sample *t*-test). Correlation (Pearson’s *r*) with the empathy concern trait was significant for the Emp scenario, but not for the Eco scenario; a similar correlation pattern was observed for helping tendency. As expected, average helping likelihood ratings were close to the midpoint of 2.5 for both the Emp and Eco scenarios, with their difference of less than 0.5. Significant correlations with both helping tendency and empathic concern were observed for the Emp scenario, but not for the Eco scenario.

**Table 1:** Behavioral data.

Rating (phase)	Scenario	Average rating (mean ± SD)			Trait correlation (*r*)			
			Emp -Eco	*p*	Empathic concern	*p*	Helping tendency	*p*
Empathic concern	Emp	3.19 ± 0.45	1.45 ± 0.39	<0.001^*^	0.48	0.004^*^	0.44	0.01^*^
(Con phase)	Eco	1.72 ± 0.27			0.30	0.09	0.31	0.07
Helping likeliness	Emp	2.22 ± 0.44	−0.41 ± 0.50	<0.001^*^	0.46	0.006^*^	0.40	0.02^*^
(Dec phase)	Eco	2.63 ± 0.38			0.08	0.67	0.24	0.17

Average rating score (range: 1–4) for the empathy dilemma (Emp) and economic-dilemma (Eco) scenarios and their difference, and their correlation coefficients (Pearson’s r) with trait scores for empathic concern (Interpersonal Reactive Index (IRI) subscale) and helping tendency (Power to Live altruism subscale), for the empathic concern rating in the empathic concern (Con) phase, and the helping likelihood rating in the helping likelihood (Dec) phase. ^*^p < 0.05 (uncorrected).

There was a moderate degree of positive correlation between the two personality traits, empathic concern and helping tendency (*r* = 0.50, *p* = 0.003).

### 4.2. fMRI results

#### 4.2.1. Confirmation of experimental manipulation

**Figure 2:**
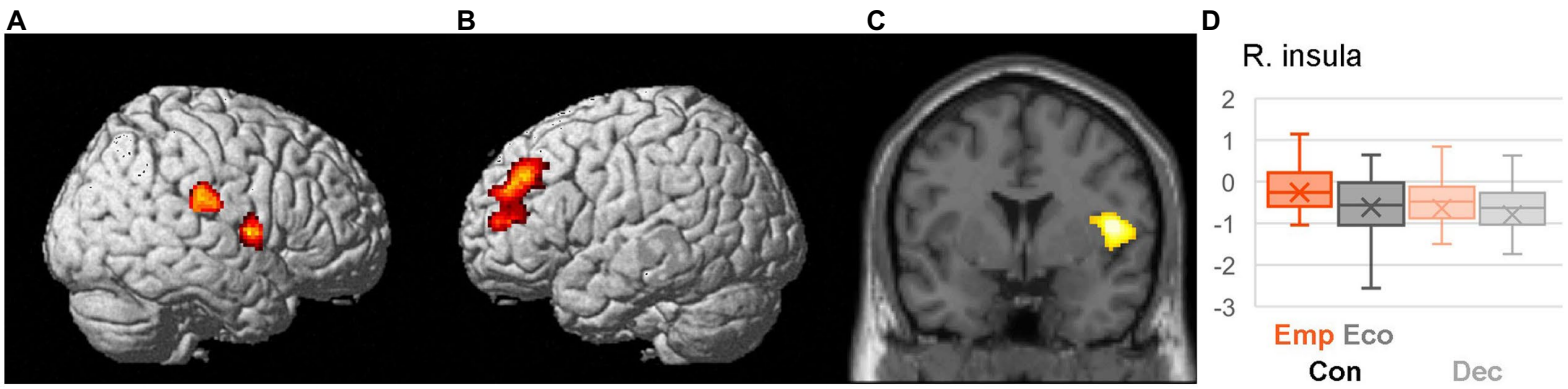
Differential activation during context presentation (EmpCon > EcoCon). Significantly higher activation during the thinking period of the context presentation for Emp than for Eco scenario (EmpCon_think > EcoCon_think) is presented in red-yellow color on the lateral surface of the right **(A)** and left **(B)** cerebral hemispheres as well as the coronal section (*y* = 2) **(C)** of the standard anatomical image of SPM12. Boxplot **(D)** shows the activation profile of the right insula; each box extends from the 25th to the 75th percentile and the middle line denotes median value of estimated activation (against baseline) during Con_think period (i.e., vs. baseline) for Emp (orange) or Eco (gray) scenarios; vertical extending line denotes adjacent values (i.e., the most extreme values within 1.5 interquartile range of the 25th and 75th percentile), and the cross mark denotes the mean.

Differential neural activation between two scenarios (Emp > Eco) during the Con phase was identified using a voxel-wise single-sample *t*-test of the contrast EmpCon_think > EcoCon_think (Table 2, Figure 2). As expected, higher activation during EmpCon was observed in the right insula and TPJ, which are implicated in empathic concern (Feldman Hall et al., 2015), suggesting our successful induction of empathic concern in the Emp scenario. Activation was also observed in the left anterior prefrontal region, including the middle frontal gyrus and inferior frontal gyrus. We performed an ROI regression analysis with the empathy concern score for each peak differential activation. We expected to find a positive correlation, which would support an association of trait empathic concern with increased activation during EmpCon trials; however, no significant effect was identified for any of the peaks (*p* > 0.05, uncorrected).

**Table 2:** Differential activation during context presentation (EmpCon > EcoCon).

Structure		Coordinate				Cluster size			
		*x*	*y*	*z*	*t*	*k*		*p*	
Insula	R	48	2	10	6.16	306		0.003	^†^
Temporoparietal junction	R	60	−22	22	4.63	318		0.008	^†^
Middle frontal gyrus	L	–30	38	36	5.50	902	a	<0.001	
Inferior frontal gyrus	L	−30	40	14	4.59		a		

For each peak voxel, laterality (L: left, R: right), Montréal Neurological Institute (MNI) coordinate, *t*-value for differential activation (EmpCon_think –EcoCon_think), and associated cluster information are provided. For each cluster, the number of voxels (*k*; 2 × 2 × 2 mm/voxel) and *p* are provided for the highest peak voxel. Identical letters indicate the same cluster. ^†^ *p* corrected according to an a priori-determined small volume.

#### 4.2.2. Neural response characterizing the altruism–egoism dilemma

**Figure 3:**
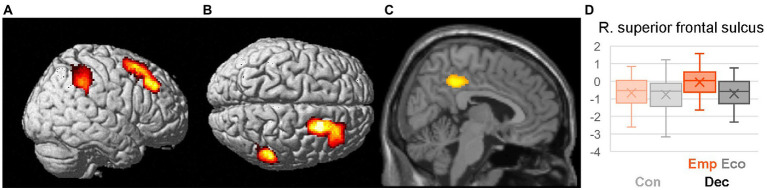
Differential activation during helping decision (EmpDec > EcoDec). Significantly higher activation during the thinking period of the context presentation for Emp than for Eco scenario (EmpDec_think > EcoDec_think) is presented on the cerebral surfaces from the right **(A)** and top **(B)** as well as on the parasagittal section (*x* = 6) **(C)**. Boxplot **(D)** shows the activation profile of the right superior frontal sulcus during Dec_think period. Other details are the same as for Figure 2.

Differential neural activation between the two scenarios (Emp > Eco) during the Dec phase was identified using a voxel-wise single-sample *t*-test of the contrast EmpDec_think > EcoDec_think (Table 3, Figure 3). Higher activation during EmpDec was observed in the right anterior prefrontal cortices, including the superior frontal sulcus and middle frontal gyrus, as well as the supramarginal gyrus and PCC, suggesting their involvement in the empathic dilemma.

**Table 3:** Differential activation during helping decision (EmpDec > EcoDec).

Structure		Coordinate				Cluster size			Helping-tendency trait effect			
		*x*	*y*	*z*	*t*	*k*		*p*	Emp	*p*	Eco	*p*
Superior frontal sulcus	R	26	12	54	6.89	1,617	a	<0.001	−1.46	0.14	−1.20	0.19
Middle frontal gyrus	R	32	34	38	5.17		a		−1.01	0.24	−1.11	0.21
Supramarginal gyrus	R	56	−46	48	6.21	554		0.012	−0.30	0.38	0.03	0.40
Posterior cingulate cortex	R	6	−40	40	4.91	370		0.046	−2.12	0.04^*^	−1.82	0.08

Differential activation (EmpDec_think –EcoDec_think) is summarized as described in Table 2. The t values for the helping tendency effect are provided for the Emp and Eco scenarios (vs. baseline) for each identified peak (20-mm spherical region of interest centered at the peak voxel). ^*^p < 0.05 (uncorrected).

#### 4.2.3. Neural correlates of the individual differences in helping tendency

**Figure 4:**
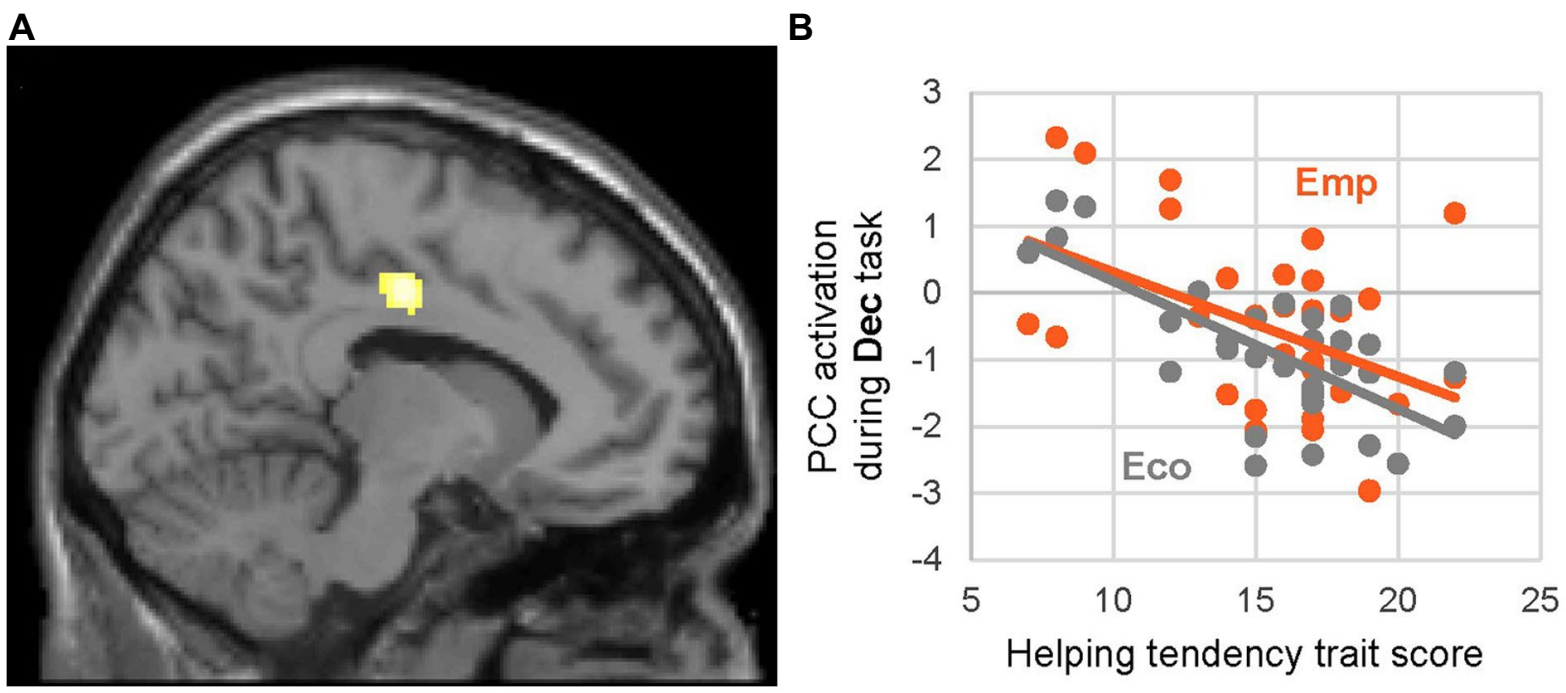
Effect of helping-tendency trait during helping decision (EmpDec + EcoDec). Significant negative effect of helping-tendency trait on activation during the thinking period on helping-decision context irrespective of the scenario type (EmpDec_think + EcoDec_think) is presented on the parasagittal section (*x* = −10) **(A)**. The scatter plot for estimated PCC [−10, −14, 40] activation during Dec_think period (i.e., vs. baseline) against the helping-tendency trait score and regression line are given separately for Emp (orange) and Eco (gray) scenarios **(B)**. Other details are the same as for Figure 2.

Our voxel-wise test of helping tendency effects on the contrast EmpDec_think -EcoDec_think, which addressed the neural response specific to empathic dilemma, showed no significant activation, whereas that of the contrast EmpDec_think + EcoDec_think (i.e., against baseline), which addressed the neural response common to both dilemma types, found a significant negative effect in the left PCC (Table 4, Figure 4). In other words, there was a negative effect of trait helping tendency in both EmpDec and EcoDec trials.

**Table 4:** Effect of helping-tendency trait during helping decision (EmpDec + EcoDec).

Structure		Coordinate				Cluster size		Helping-tendency trait effect			
		*x*	*y*	*z*	*t*	*k*	*p*	Emp	*p*	Eco	*p*
Posterior cingulate cortex	L	−22	−16	40	−4.44	454	0.008	−3.00	0.006^*^	−3.60	0.001^*^
Posterior cingulate cortex	L	−10	−14	40	−4.32			−2.68	0.01^*^	−5.39	<0.001^*^

Significant negative effect of helping tendency on activation during the thinking period during a helping decision, irrespective of the scenario type (EmpDec_think + EcoDec_think). The results are summarized as described in Table 3.

*Post-hoc* ROI analyzes for the activation peaks identified in this analysis (Table 4) detected a significant negative trait effect for both scenarios (i.e., EmpDec_think and EcoDec_think against baseline); these effects were not significant for the EmpDec_think –EcoDec_think contrast, suggesting a negative effect of trait helping tendency in both scenarios. Among the activation peaks identified in the voxel-wise single-sample *t*-test of differential activation during the helping decision contrast (i.e., EmpDec_think > EcoDec_think), a negative effect was detected at the right PCC under the Emp scenario, whereas those under the Eco scenario and difference (Emp –Eco) were not significant (Table 3). Therefore, there was no clear evidence of scenario-specific versus general effects.

## 5. Discussion

Using context-rich scenarios to allow contextual elaboration for constructing reasons to overcome the moral pain associated with not helping a disadvantaged person, we explored the neural responses characterizing naturalistic altruism–egoism dilemmas. We detected activation of the right anterior prefrontal cortices, supramarginal gyrus, and PCC during the decision as to whether to help under an empathy dilemma (i.e., EmpDec > EcoDec). Consistent with our expectation, these identified regions largely overlapped with the cortical areas implicated in moral dilemmas (Moll et al., 2002, 2006; Greene et al., 2004; Reniers et al., 2012), supporting commonality between altruism–egoism and moral dilemmas. Among these regions, the PCC showed a significant negative effect of helping tendency and neural response during EmpDec, suggesting greater involvement of this region in people who tend to help less in daily life. A more robust negative effect of this trait was detected in the left PCC by its close proximity in the voxel-wise search; interestingly, this negative effect was observed during both the EmpDec and EcoDec periods.

The identified neural correlates of altruism–egoism dilemmas appear to be related to the egoistic counter-dynamics of helping decisions in naturalistic context-rich situations. Anatomical overlap was also prominent in context-rich studies of decision-making in the context of moral dilemmas (Greene et al., 2004; Reniers et al., 2012), as well as in studies that analyzed responses to morality-related emotional images (Moll et al., 2002) and asked participants to make donations to organizations without providing any context (Moll et al., 2006). These studies reported involvement of the mesolimbic and orbitofrontal cortices in decision-making, which was not detected in the current study. Studies in which a moral dilemma was embedded in the context of decision-making (Greene et al., 2004; Reniers et al., 2012) reported activation of the supramarginal gyrus and PCC, consistent with our results. These regions are activated during the evaluation of realistic situations for which there is no obviously correct choice, such as between two roads (to reach one’s place of employment; familiar congested road vs. newly constructed shortcut) (Pearson et al., 2011), as well as during self-evaluation of emotions after being offered help with luggage by a stranger (Olivo et al., 2021). Therefore, these areas may be involved in contextual elaboration as it pertains to justifying decisions, regardless of the decision type. The supramarginal gyrus, which plays a role in processing action goals (Van Overwalle and Baetens, 2009), and the PCC, which is involved in autobiographical information processing (Leech and Sharp, 2014; Busler et al., 2019), may be important for contextual elaboration in realistic situations; these areas are also known to play a role in episodic simulation of future events (Schacter et al., 2007, 2008). The anterior prefrontal cortices, which have been implicated in moral dilemmas irrespective of contextual richness (Moll et al., 2006; Schaich Borg et al., 2006; Reniers et al., 2012), play a general role in cognitive control and may also be involved in dilemma resolution (de la Vega et al., 2016).

The role of PCC activation in the egoistic counter-dynamics of helping decisions may be conceptualized around the comparison and integration of different types of values, considering its anatomical location and relationship with behavioral data. Anatomically, the PCC and adjacent precuneus are considered to have dorsal and ventral functional subdivisions (Leech et al., 2011; Stawarczyk and D’Argembeau, 2015); in both of these studies, PCC activation occurred at the dorsal subdivision. In the context of decision-making, activation of the dorsal subdivision was observed during choices between fixed amounts of money and the probability of winning incommensurable goods such as food (Fitz Gerald et al., 2009), and higher activation was associated with better evaluation performance in people or consumer products based on 12 attributes (Kageyama et al., 2019). There appears to be a gap between the behavioral and neural data, as the effect of helping tendency on helping likelihood was specific to the Emp scenario in this study, whereas its effect on neural activation during decision-making was common to both scenario types. Thus, in people who tend to help less in daily life, PCC activation was high during the decision as to whether to help under both empathic and economic dilemmas, and was behaviorally reflected in reduced helping likelihood only under the former condition. This gap may occur because the participant must compare and integrate altruistic (i.e., socioemotional) and egoistic (i.e., materialistic) values under the empathic dilemma (Volz et al., 2017; Lee et al., 2019), whereas competing values are largely egoistic under the economic dilemma.

The negative effect of helping tendency on PCC activation observed in this study appears consistent with the typically inverse relationship between adaptive personality traits and the degree of neural activity. Helping tendency is considered to be an adaptive personality trait; on the Power to Live questionnaire, as one of the eight psychobehavioral characteristics associated with surviving disasters, this trait is scored using the altruism subscale (Sugiura et al., 2015), and showed associations with both the tendency to help others (Sugiura et al., 2020) and be helped by others in the aftermath of a disaster (Sugiura et al., 2021). These findings are consistent with the notion that altruism is an adaptive trait in social processes, although the evolutionary process remains controversial (Trivers, 1971; Glassman, 2000; Boyd and Richerson, 2009). Intuitively, the adaptive capacities or abilities are generally achieved through increased brain activity. However, adaptive personality traits are rarely associated with higher brain activation in situations where the adaptive nature of the trait is exerted. For example, among other Power to Live subscales, the adaptive trait of problem solving is associated with lower brain activation in motor-related areas (Miura et al., 2020a), stubbornness (i.e., resistance to social conformity pressure) in a cognitive control area (Miura et al., 2020b), and emotion regulation in extensive cortical regions including the prefrontal control system (Sugiura et al., 2023). The concept of mindfulness (Bishop et al., 2004) may be relevant to adaptive reductions of PCC activity. Trait mindfulness was correlated with prosocial behavior (Donald et al., 2019) and reduced PCC activation was observed during mindful acceptance of emotions (Messina et al., 2021). However, the relationship between PCC activation and adaptability may be nonlinear; fear of death and PCC activation exhibited a quadratic relationship during the contemplation of one’s own death (Hirano et al., 2021).

## 6. Conclusion and implications

### 6.1. Conclusion

Based on these considerations, we propose a two-stage model of altruistic helping decision-making. The first stage is an altruism–egoism dilemma process, in which empathy-driven helping motivation conflicts with egoistic cost and may be subject to little individual difference. This stage may feature a contextual elaboration process for constructing reasons for the decision in naturalistic context-rich situations. Our data suggest involvement of the right supramarginal gyrus and anterior prefrontal cortices in this stage, and we suggest that they have roles in sensorimotor aspects of contextual elaboration and cognitive control for dilemma resolution, respectively. The second stage concerns the key counter-dynamics of the helping decision, and is responsible for individual differences in the helping tendency in daily life. This stage may be related to the comparison and integration of different types of values relevant to episodic simulation of future events based on autobiographical information.

### 6.2. Theoretical implications

There are two important implications of this proposed model. First, the important determinant of individual differences in the helping tendency appears to exist outside of the empathic process, in contrast to the classic view (Batson et al., 1991; Feldman Hall et al., 2015). Second, the latter stage appears to be non-specific to altruistic helping decisions; although the effect was expressed behaviorally only in altruistic helping in this study, the effect of this trait on the neural process was also present in the egoistic helping decision and may be expressed behaviorally in other decision contexts in daily life. Support for this notion is provided by the finding of PCC involvement in maladaptive decision-making in the context of attention-deficit/hyperactivity disorder (Sonuga-Barke and Fairchild, 2012) and HIV infection (Hall et al., 2021).

### 6.3. Social implications

Our study highlights the altruism–egoism dilemma and counter-dynamic processes in social helping decisions, a process that previous studies have described as complex (Sanfey, 2007; Ramsøy et al., 2014). In conclusion, the results of this study provide a social understanding of how helping decisions are made by balancing the costs and benefits according to oneself, and why some people do help while others do not.

## 7. Study limitations

There were several limitations to this study. First, the helping decisions were “virtual;” thus, our findings may not extend to helping decisions with real-world consequences. However, we believe that the psychological and neural processes stimulated by the tasks in this study accurately reflect those stimulated by real-world helping decisions; the helping likelihood rating was correlated with helping tendency, which was previously shown to be associated with real-world helping behaviors (Sugiura et al., 2020). Second, with respect to our conceptualization and experimental manipulation of the empathic process, we did not explicitly take into account a multidimensional model that includes perceptual and cognitive components of empathy (Gallese, 2003; Innamorati et al., 2019). However, the empathy-related brain regions identified in the current study (i.e., the insula and TPJ, as well as prefrontal areas) partly overlap with the putative neural correlates of perceptual and cognitive components of empathy (Ebisch et al., 2022).

## Data availability statement

The raw data supporting the conclusions of this article will be made available by the authors, without undue reservation.

## Ethics statement

The studies involving human participants were reviewed and approved by Tohoku University School of Medicine Ethics Committee (2018-1-785). The patients/participants provided their written informed consent to participate in this study.

## Author contributions

VW, KO, RI, and MS contributed to the conception and design of the study and analyzed the data. KO, RI, and VW prepared the experimental stimuli and carried out the experiment. VW and MS wrote the manuscript. All authors contributed to the manuscript and approved the submitted version.

## Funding

This study was supported by the Grant-in-Aid for Scientific Research on Innovative Areas (Research in a proposed research area; KAKENHI 22H04855) from the Japan Society for the Promotion of Science to MS.

## Conflict of interest

The authors declare that the research was conducted in the absence of any commercial or financial relationships that could be construed as a potential conflict of interest.

## Publisher’s note

All claims expressed in this article are solely those of the authors and do not necessarily represent those of their affiliated organizations, or those of the publisher, the editors and the reviewers. Any product that may be evaluated in this article, or claim that may be made by its manufacturer, is not guaranteed or endorsed by the publisher.
